# Methanolic Extract of* Artemia salina* Eggs and Various Fractions in Different Solvents Contain Potent Compounds That Decrease Cell Viability of Colon and Skin Cancer Cell Lines and Show Antibacterial Activity against* Pseudomonas aeruginosa*

**DOI:** 10.1155/2019/9528256

**Published:** 2019-05-06

**Authors:** Salman Ul Islam, Muhammad Bilal Ahmed, Adeeb Shehzad, Young Sup Lee

**Affiliations:** ^1^School of Life Sciences, College of Natural Sciences, Kyungpook National University, 41566, Republic of Korea; ^2^Department of Clinical Pharmacy, Institute for Research and Medical Consultations (IRMC), Imam Abdurahman Bin Faisal University, Dammam, Saudi Arabia

## Abstract

*Artemia salina,* crustaceans of class Branchiopoda and order Anostraca, are living and reproducing only in highly saline natural lakes and in other reservoirs where sea water is evaporated to produce salt.* Artemia salina* eggs can be purchased from pet stores, where they are sold as tropical fish food and a ready source for hatching shrimp. In the current study, methanolic crude extracts and various fractions of* Artemia salina* eggs extracted in other solvents were tested for effects on cell viability of human colorectal cancer cells (HCT116) and melanoma cells (B16F10) using an MTT (3-(4,5-dimethylthiazol-2-yl)-2,5-diphenyltetrazolium bromide) assay. A methanolic crude extract of eggs was obtained by cold maceration, followed by fractionation to obtain hexane, chloroform, ethyl acetate, n-butanol, and aqueous fractions. The methanolic crude extract decreased cell viability of HCT-116 and B16F10 cell lines at higher concentrations. The other fractions were evaluated using a cell viability assay, and chloroform and hexane showed the highest activity at significantly lower concentrations than did the methanolic fraction. Full scan profiles of the methanolic crude extract and the chloroform and hexane fractions were obtained by gas chromatography mass spectrometry (GC-MS), and the resultant compounds were identified by comparing their spectral data to those available in spectral matching libraries. ROS generation assay, flow cytometry, and western blot analysis provided supporting evidence that the hexane and chloroform fractions induced cell death in HCT116 and B16-F10 cell lines. All fractions were further tested for antibacterial activity against* Pseudomonas aeruginosa*, among which the hexane fraction showed the highest zone of inhibition on LB nutrient agar plates. This study demonstrated promising anticancer and antibacterial effects of* Artemia salina* egg extracts. Our results suggest that pure bioactive compounds obtained from* Artemia salina* eggs can provide new insights into the mechanisms of colon and skin cancer, as well as* Pseudomonas aeruginosa* inhibition.

## 1. Introduction

The popularity of natural products as chemopreventive substances is increasing steadily because of their potential effectiveness and low toxicity [1]. Recently, marine fauna and flora have received significant attention as potent sources of novel chemopreventive agents. High potency antitumor agents have been discovered in marine sources [2]. Strong anticancer activities have been shown in extracts from algae, sponges, and marine cyanobacteria [3–5]. Fucoidans, alginic acids, laminarans, and carrageenans are among the marine-based compounds that exert potent anticancer activities. In addition, miscellaneous polysaccharides extracted from marine animals, fungi, and bacteria have been identified as potential anticancer agents, many of which have been evaluated for further drug development [3]. Anticancer drugs of marine origin available commercially include cytarabine, trabectedin, eribulin mesylate, and brentuximab vedotin [6, 7]. Many other marine derived substances with potential anticancer activity are currently being investigated in preclinical studies [5, 8].

*Artemia salina*, also known as brine shrimp, live in highly saline natural lakes, such as the Great Salt Lake in northern Utah and the Caspian Sea, on the rocky coast to the south of San Francisco, and in man-made evaporation ponds used to produce salt from the ocean. They show a remarkable resistance to change and can survive in water with a wide range of salinity. Salty locations are favorable for* Artemia salina* because there are few predators, but food is also limited in these environments [9].* Artemia salina* are used in toxicity assays and for various other academic purposes, because they reproduce rapidly and their natural habitat can be easily replicated. Owing to their low cost and ease of use, adult* Artemia salina* and their eggs are utilized to feed coral, larval fish, and other crustaceans.* Artemia salina* and their eggs can also be enriched with proteins, lipids, and other nutrients beneficial to the animal consuming them. Additionally, the nauplii (larvae) can also serve as a carrier of therapeutic substances to treat or prevent diseases [10–12].

Multiple reports are available on the medicinal potential of different marine species and their eggs. For instance, a study demonstrated that hairtail egg, Spanish mackerel egg, and Pacific saury egg contained lysophosphatidic acid and lysophosphatidylcholine, with saturated or unsaturated acyl chains as major lysophospholipid inhibitors, because of which these eggs showed a strong inhibition of lysophospholipase D activity [13]. Studies have shown that omega-3 and omega-6 fatty acids (components of lipid fractions extracted from fish) are associated with the prevention of cardiovascular diseases and cancer [14–16]. Similarly, a study reported that a hexapeptide, Phe-Ile-Met-Gly-Pro-Tyr (FIMGPY), extracted from skate (*Raja porosa*) cartilage protein hydrolysate, exhibited antiproliferation activities in HeLa cells [17]. To date, however, there are no reports on the medicinal values of* Artemia salina* eggs.

In this study we analyzed the bioactive compounds present in* Artemia salina* eggs and investigated their activity against colorectal and skin cancer cell lines and* Pseudomonas aeruginosa*, which is the most important causative bacterium of burn-associated infections.

## 2. Materials and Methods

### 2.1. Chemicals and Reagents

Methyl alcohol (product no. 62), n-hexane (product no. 4794), ethyl acetate (product no. 2936), and chloroform (product no. 1268) were purchased from Duksan chemicals, South Korea. 1-butanol (cat# 33065) was purchased from Honeywell research chemicals. 5-Fluorouracil (cat# F6627), propidium iodide (PI), and 3-(4,5-dimethylthiazol-2-yl)-2,5- diphenyltetrazolium bromide (MTT) were obtained from Sigma-Aldrich (St. Louis, MO, USA). Dihydrorhodamine 123 (DHR 123: cat# D23806) was purchased from ThermoFisher scientific. Antibodies [cleaved caspase-3 (cat# sc-22171), cleaved PARP (cat# sc-56196), and *β*-actin (cat# sc-47778)] were purchased from Santa Cruz Biotechnology (Santa Cruz, CA, USA). 4′,6-diamidino-2-phenylindole (DAPI) mounting solution (Cat# H-1200) was purchased from Vector laboratories, USA. All the chemicals and reagents were used as directed by the manufacturers.

### 2.2. Preparation of Samples

Pure* Artemia salina* eggs were commercially obtained from Ocean Nutrition™ (https://www.oceannutrition.com). The eggs were macerated in pure methanol for 48-72 h. The extraction procedure was repeated three times and the extracts were combined. Methanol was evaporated using a rotary evaporator under reduced pressure to obtain a dried methanolic extract of the eggs. A portion of the methanolic extract was resuspended in methanol and fractionated into solvents with successively increasing polarity. Hexane, chloroform, ethyl acetate, n-butanol, and residual aqueous fractions were obtained.

### 2.3. Cell Culture

Two cancer cell lines, human colorectal carcinoma cells (HCT116: ATCC® CCL-247™) and mouse skin melanoma cells (B16-F10: ATCC® CRL-6475™), were used in the current study. The cells were cultured in Dulbecco's Modified Eagle's Medium (DMEM) with 10% fetal bovine serum (FBS), L-glutamine, and 1% (v/v) penicillin-streptomycin (Gibco, USA) and maintained at 37°C, 5% CO2, and 95% humidity [18].

### 2.4. Cell Viability Assay

Cell viability was measured by the MTT assay as previously reported previously [19, 20]. Cells were seeded into 96-well plates at a density of 2 × 10^5^ cells/well. Triplicate wells were separately treated across a range of concentrations (0, 5, 10, 20, 40, 80, 100, and 250 *μ*g/ml) of* Artemia salina* egg crude extract or partially purified fractions. After incubation at 37°C with 5% CO_2_, the medium was removed and replaced with 200 *μ*l of fresh medium with 20 *μ*l of 5 mg/ml MTT solution and incubated for 4 h. The medium with MTT was removed and 200 *μ*l of DMSO was added to each well. The plates were then gently agitated until the reaction was uniform in color. OD540 (optical density at 540 nm) was determined using a 96-well plate reader. Control cells served as an indicator of 100% cell viability.

### 2.5. GC-MS Analysis

GC-MS analysis of the methanol extract and other fractions of* Artemia salina* eggs was performed using an Agilent system (7890B-5977B GC/MSD with column; J & W 122-5532DB-5MS). GC-MS conditions used for analysis and identification of* Artemia salina* egg extracts are summarized in Supplementary Table (available here). The separated compounds were analyzed by GC-MS and retention times for all compounds were determined. The compounds were identified based on comparison of their mass spectra with those of the internal (computer) library W9N11.L.

### 2.6. Antibacterial Assay

*Pseudomonas aeruginosa* (Carolina, USA, cat# 155250A) strains were revived from frozen stocks stored at -80°C by streaking on LB media (Difco LB-Miller cat#244620) plates and incubation overnight at 37°C. Screening of the ethyl acetate, hexane, chloroform, water, and n-butanol fractions for antibacterial activity against* Pseudomonas aeruginosa* was performed using the paper disc method [21]. Fifty microliters of 2 *μ*g/*μ*l stock solutions of the extracts were slowly absorbed into the sterilized paper disc and adhered to the surface of the plate on which 10^6^ CFU/ml* Pseudomonas aeruginosa* had been inoculated. 1% silver sulfadiazine was used as a standard. After culturing for 24 h in an incubator at 37°C, the clear zones around the disks were measured in millimeters and the antibacterial activities were analyzed and compared. Analysis was performed in triplicate, and the results were reported as the mean ± SD.

### 2.7. Mitochondrial ROS Generation Assay

Intracellular ROS was detected using dihydrorhodamine 123 (DHR123), which is an uncharged and nonfluorescent ROS indicator that can passively diffuse across membranes where it is oxidized to cationic rhodamine 123, which localizes to mitochondria and exhibits green fluorescence [22]. To detect ROS generation after treating cells with the hexane and chloroform fractions, 10 *μ*M DHR123 was added to the cell culture medium for 20-30 min in the dark. The cells were washed with PBS and nuclei were counterstained with DAPI mounting solution for 5–10 minutes. Cells were then analyzed using a confocal laser scanning platform (DM/R-TCS, Leica) coupled to a microscope (Leitz DM REB).

### 2.8. Flow Cytometry

Cells were treated with the hexane and chloroform fractions for 24 h. Cells were harvested, washed twice with cold PBS, and fixed with 75% ethanol at −20°C overnight. After fixation, the cells were washed with cold PBS and incubated in staining buffer (50 *μ*g/ml PI and 1 mg/ml RNase) at 37°C in the dark for 30 min. Subsequently, the samples were analyzed using a flow cytometer (BD Biosciences, San Diego, CA, USA). The percentages of cells in the G_0_/G_1_, S, and G_2_/M phases were calculated using Cell Quest acquisition software (BD Biosciences). Analysis was performed in triplicate, and the results were reported as the mean ± SD [23].

### 2.9. Western Blotting

Cells were collected using a cell scraper in PBS and centrifuged at 12,000 rpm for 5 min to obtain cell pellets. The supernatant were discarded and the cell pellets were resuspended in 200 *μ*l of cell lysis buffer (50 mM Tris, pH 7.4, 0.5% NP40, 0.01% SDS, and protease inhibitor cocktail (Roche, Germany)). After lysis by sonication, total protein in cell lysates was quantified using the Bio-Rad Protein Assay according to the manufacturer's protocol. Samples (20–40 *μ*g) were prepared in SDS sample buffer containing 60 mM Tris-HCl (pH 6.8), 2% SDS, 10% glycerol, and 5% *β*-mercaptoethanol, then separated on a 10–12% SDS-PAGE gel, and transferred onto a polyvinylidene fluoride (PVDF) membrane (Amersham, Piscataway, NJ, USA). The membranes were blocked with 3% albumin (Gendepot, USA) solution for 2 h at 4°C. Chemiluminescent signals were developed using Clarity™ ECL Western Blotting Substrate (Bio-Rad) according to the manufacturer's instructions [18, 24].

### 2.10. Statistical Analysis

Unless stated otherwise, statistical significance was determined by one-way analysis of variance based on three independent experiments. Differences were considered statistically significant at p < .05.

## 3. Results and Discussion

### 3.1. Effect of Artemia salina Egg Extracts on HCT116 and B16-F10 Cell Viability

**Figure 1:**
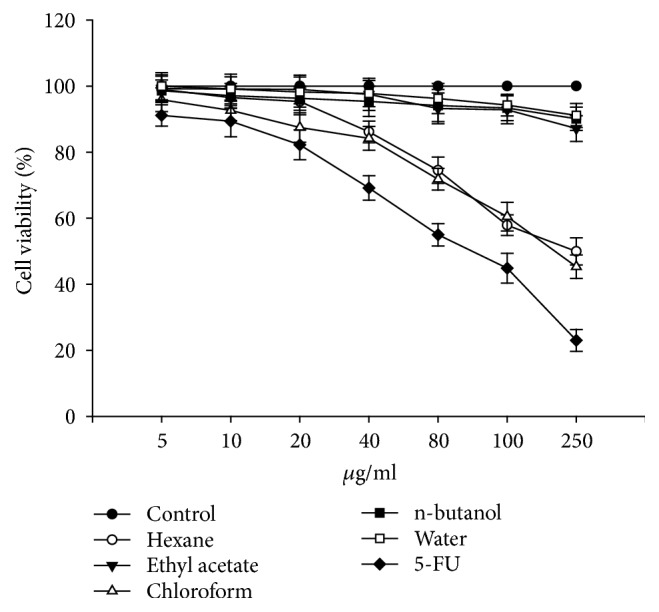
Effect of* Artemia salina* egg extracts on HCT116 cell viability. Triplicate wells were treated across a range of extract concentrations (0, 5, 10, 20, 40, 80, 100, and 250 *µ*g/ml) for 24 h. 5 fluorouracil (5-FU) was used as standard. Control cells served as an indicator of 100% cell viability. Hexane and chloroform fractions exhibited the greatest activity.

**Figure 2:**
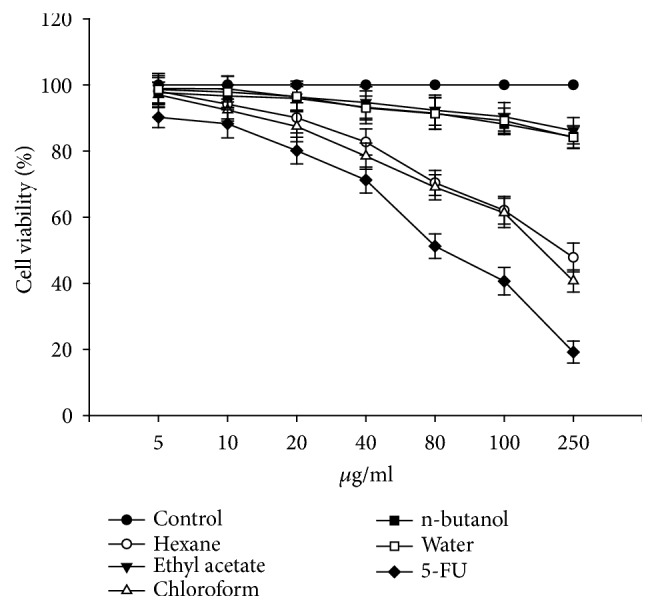
Effect of* Artemia salina* egg extracts on B16-F10 cell viability. Cells were treated in triplicate cross a range of extract concentrations (0, 5, 10, 20, 40, 80, 100, and 250 *µ*g/ml) for 24 h. 5-FU was used as standard. Control cells served as an indicator of 100% cell viability. Hexane and chloroform fractions exhibited the greatest activity.

To analyze the effect of* Artemia salina* egg extracts on the viability of HCT116 and B16-F10 cells, we first treated the cells with methanolic crude extracts. Methanolic crude extracts decreased the viability of HCT116 and B16-F10 cells by up to 50% at concentrations ranging from 500 to 600 *μ*g/ml (data not shown). We further incubated the cells for 24 h with partially purified fractions (hexane, ethyl acetate, chloroform, n-butanol, and water) at increasing concentrations (0, 5, 10, 20, 40, 80, 100, and 250 *μ*g/ml) and observed that hexane and chloroform fractions remarkably reduced cell viability of both cells lines. 5 fluorouracil (5-FU) was used as standard. Treatment with 250 *μ*g/ml of the hexane and chloroform fractions reduced cell viability of HCT116 cells to 49.94% and 45.31%, respectively (Figure 1). Treatment of B16-F10 cells with 250 *μ*g/ml of the hexane and chloroform fractions reduced cell viability to 47.79% and 40.68%, respectively (Figure 2). The other fractions evaluated did not reduce cell viability of either cell line. For example, 250 *μ*g/ml of the aqueous fraction resulted in only a 15.87% reduction in B16-F10 cell viability (Figure 2). These cell viability data showed that the hexane and chloroform fractions effectively reduced cell viability of the HCT116 and B16-F10 cell lines, indicating the presence of bioactive compounds in these fractions, which could be further purified using more intricate extraction techniques.

### 3.2. Identification of Chemical Constituents

**Figure 3:**
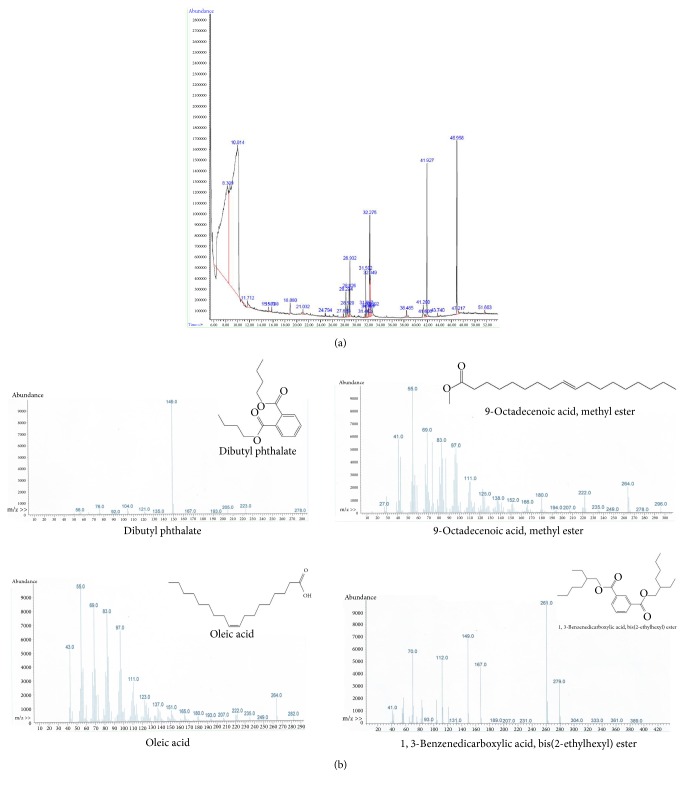
GC-MS analysis of methanolic crude extract of* Artemia salina* eggs. (a) Chromatogram of the methanolic crude extract of* Artemia salina* eggs. (b) Compounds identified in the methanolic crude extract of* Artemia salina* eggs. Details of these compounds are summarized in Table 1.

**Figure 4:**
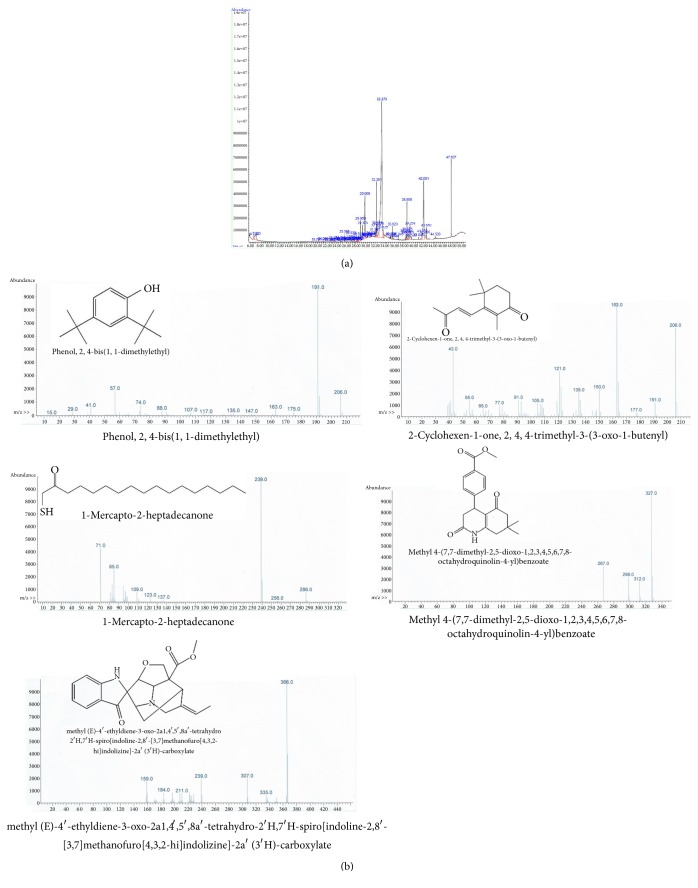
GC-MS analysis of the hexane fraction of the methanolic crude extract. (a) Chromatogram of the hexane fraction of the methanolic crude extract. (b) Compounds identified in the hexane fraction of methanolic crude extract. Details regarding these compounds are summarized in Table 1.

**Figure 5:**
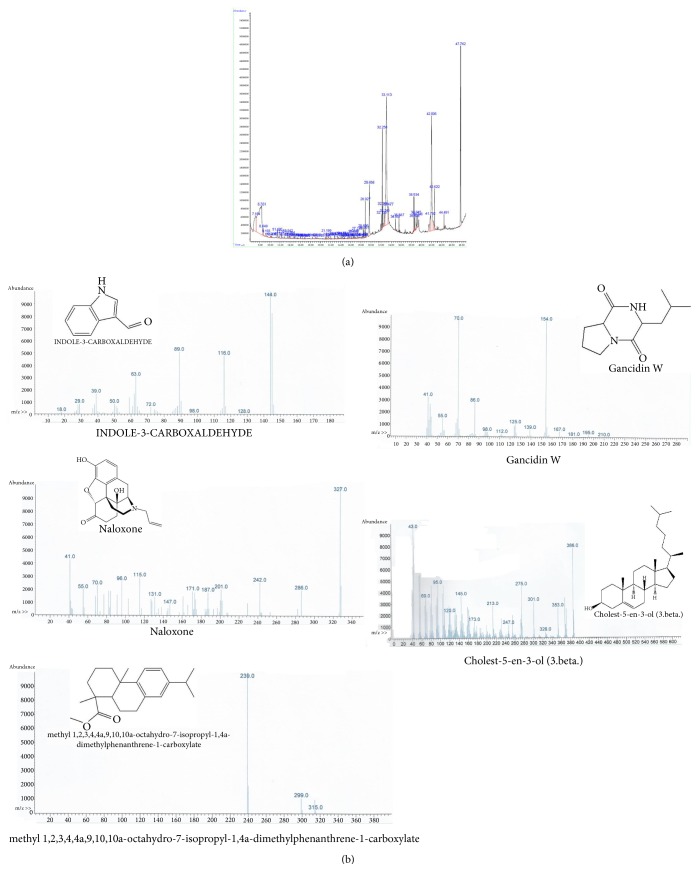
GC-MS analysis of the chloroform fraction of the methanolic crude extract. (a) Chromatogram of the chloroform fraction of the methanolic crude extract. (b) Compounds identified in the chloroform fraction of the methanolic crude extract. Details regarding these compounds are summarized in Table 1.

GC-MS analysis was used to identify bioactive compounds in* Artemia salina* egg methanolic crude extract and hexane and chloroform fractions [25, 26]. Hexane and chloroform fractions were chosen for compound identification based on cell viability experiment results. GC-MS analysis of the methanolic extract of* Artemia salina* eggs showed several peaks which indicated the presence of chemical constituents (Figure 3(a)). Comparison of the mass spectra of the constituents with those in the internal (computer) library W9N11.L resulted in identification of four chemical constituents: dibutyl phthalate, 9-octadecenoic acid methyl ester, Oleic acid, and di(2-ethylhexyl)phthalate (Figure 3(b)). Dibutyl phthalate has been previously reported to exert anticancer activity against lung adenocarcinoma (SPC-A-1) cells and human papillomavirus-related endocervical adenocarcinoma (BEL-7402) cells [27]. Another study reported that dibutyl phthalate inhibited growth of human lung carcinoma (A549) cells [28]. Furthermore, dibutyl phthalate showed significant activity against the gram negative bacteria* Klebsiella pneumonia*,* Proteus mirabilis*, and* Pseudomonas aeruginosa* at a concentration of 40 *μ*L/m [29]. 9-octadecenoic acid methyl ester has been shown to exert antioxidant and anticancer activities [30–32]. Several studies have reported that oleic acid reduced proliferation of prostate carcinoma (PC-3) cells [33]. In addition, oleic acid can induce* Staphylococcus aureus* death through a mechanism involving bacterial lipids [34]. Studies have shown that different staphylococci species are not capable of metabolizing oleic acid and that oxidation products of oleic acid are highly toxic to bacterial cells [35, 36]. Di(2-ethylhexyl)phthalate has been reported to exert antileukemic activity as evidenced by growth inhibition in three human leukemic cell lines, K562, HL60, and U937, at a low concentration [37]. Five major compounds with previously established bioactivity were identified in the hexane (Figures 4(a) and 4(b)) and chloroform (Figures 5(a) and 5(b)) fractions. These compounds were previously shown to exert antimycobacterial, antitumor, opioid receptor antagonistic, antifungal, anti-inflammatory, and antimalarial activities [38–45]. For example, 2,4-bis(1, 1-dimethylethyl)phenol has been shown to effectively control biofilms of* Serratia marcescens* [43]. Indole-3-carboxaldehyde efficiently inhibited human liver carcinoma (HepG2), human breast adenocarcinoma (MCF7), human ductal breast epithelial tumor (T47D), A549, human cervix adenocarcinoma (HeLa), and mouse fibroblast (L929) cells with considerable selectivity [38]. Moreover, a study evaluated the effect of naloxone on human breast cancer cell growth and progression in a mouse model of human triple-negative breast cancer generated by injecting MDA.MB231 (estrogen receptor-negative human breast carcinoma cells subcutaneously into mice. This study demonstrated proliferation of MDA.MB231 cells was inhibited, and cell death increased, in a dose-dependent manner in response to naloxone.* In vivo* studies showed that tumors in mice treated with naloxone were significantly smaller than those observed in the control groups [40]. Complete details of all identified compounds along with their activities are summarized in Table 1. The presence of various bioactive compounds in* Artemia salina* eggs justifies their potential use for treatment of various ailments. However, isolation of individual chemical constituents and subsequent evaluation of biological activity will allow for further characterization of therapeutic potential of* Artemia salina* egg extracts. Based on our results,* Artemia salina* eggs contain various bioactive compounds which may be of pharmaceutical importance.

### 3.3. Apoptosis-Inducing Potential

**Figure 6:**
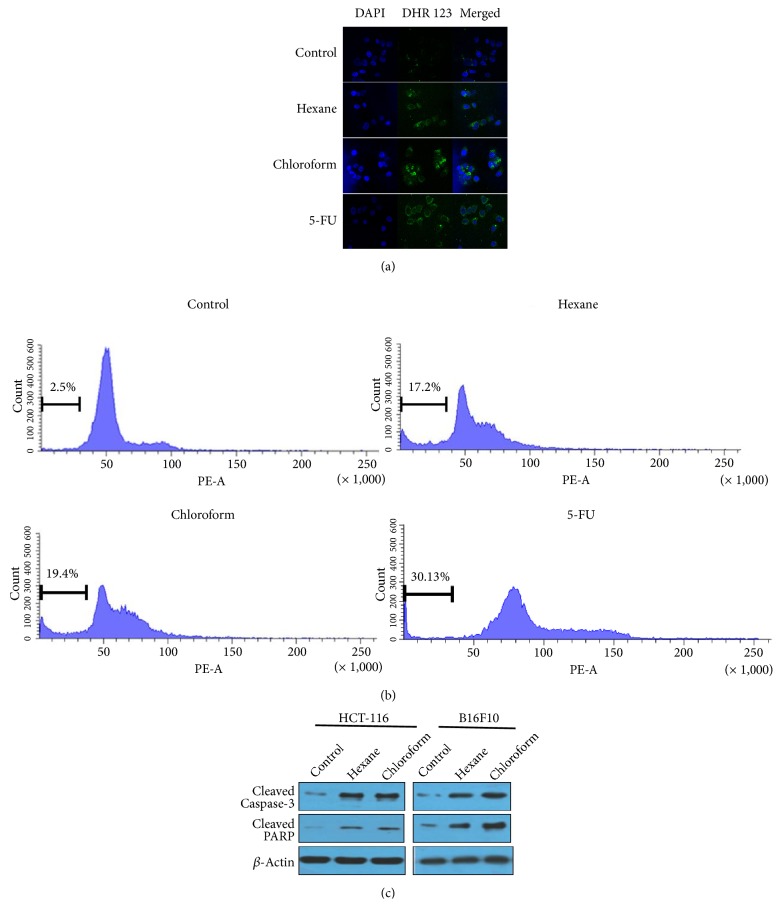
*Hexane and chloroform fractions of the methanolic extract induced apoptosis in HCT116 and B16-F10 cells*. (a) Intracellular ROS generation was determined by treating cells with 100 *µ*g/ml hexane and chloroform fractions for 24 h and then incubating them with DHR123 for 20-30 min. Images were obtained using a fluorescence microscope, with excitation and emission wavelengths of 500 and 536 nm, respectively. 5-FU was used as standard. (b) Cells were treated with 10 *μ*g/ml hexane and chloroform fractions. Cells were collected, fixed with 70% ethanol overnight at −20°C, and then stained with PI. DNA content was analyzed by flow cytometry. 5-FU was used as standard. (c) Cellular proteins were extracted using cell lysis buffer. Proteins were quantified by Bradford assay, and equal amount of proteins was loaded onto SDS-PAGE gels. Electrophoresis was conducted at a constant voltage of 100V for 140 min. Proteins were then transferred onto nitrocellulose (NC) membranes. NC membranes were blocked with 3% BSA for 1 h at room temperature. NC membranes were then incubated with specific antibodies (1:1000) for cleaved caspase-3 and cleaved PARP overnight at 4°C. NC membranes were washed with TBST for 40 min and then incubated with HRP-conjugated anti-rabbit or anti-mouse secondary antibodies (1:2000). Chemiluminescent signals were developed using Clarity™ ECL Western Blotting Substrate. Actin was used as internal control in all experiments.

As we observed the presence of previously reported anticancer agents in* Artemia salina* egg extracts, we hypothesized that these extracts could induce apoptosis in cancer cell lines. Furthermore, our results in this study showed that the hexane and chloroform fractions of* Artemia salina* egg extracts potently decreased viability of HCT116 and B16-F10 cells. Reactive oxygen species (ROS) generation is related to induction of apoptosis in cancer cells [46, 47]. As such, we analyzed ROS generation using a DHR 123 probe after treating cells with either the hexane or chloroform fractions. 5-FU was used as standard. Compared to untreated cells, increased rhodamine 123 fluorescence was observed in hexane and chloroform fraction-treated cells (Figure 6(a)). Next, we incubated cells with 100*μ*g/ml of hexane and chloroform fractions for 24h and then measured the sub-G_1_ fraction from fixed nuclei by PI staining and flow cytometry. As shown in Figure 6(b), the hexane and chloroform fractions increased the proportion of cell death (17.2% and 19.4% respectively) at the sub-G_1_ phase of the cell cycle. Western blot analysis was performed to determine caspase-3 activation in cells treated with the hexane and chloroform fractions. We observed a reduction of the 32kDa caspase-3 zymogen, an increase in the p11 subunit of caspase-3, and increased cleaved PARP. These results indicated that caspase-3 was activated in HCT116 and B16-F10 cells in response to treatment with the hexane and chloroform fractions (Figure 6(c)). Collectively, these results suggest that the hexane and chloroform fractions induced oxidative stress-induced apoptosis in HCT116 and B16-F10 cells through activation of caspase-3.

### 3.4. Antibacterial Activity against Pseudomonas aeruginosa

**Figure 7:**
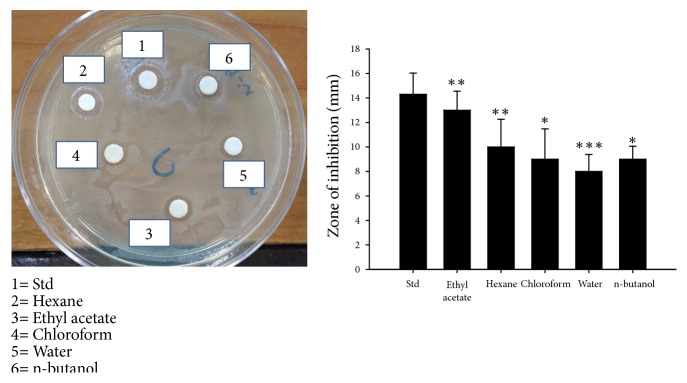
*Antibacterial activity against Pseudomonas aeruginosa*.* Pseudomonas aeruginosa* (10^6^ CFU/ml) was cultured on LB agar plates. Fifty microliters of 2 *µ*g/*µ*l stock solutions of the hexane and chloroform extracts was slowly absorbed into the sterilized paper disc (diameter: 8 mm) and adhered to the surface of the plate. One percent silver sulfadiazine was used as a standard. The clear zone around the disk was measured in millimeters. Analysis was performed in triplicate, and the results were reported as the mean ± SD. *∗*P < 0.05; *∗∗*P < 0.01; *∗∗∗*P < 0.01.

Using GC-MS analysis, we observed various compounds in* Artemia salina* eggs such as dibutyl phthalate, oleic acid, and phenol, 2, 4-bis(1, 1-dimethylethyl), for which antibacterial activity had previously been reported (Table 1). Based on these results, we evaluated activity of* Artemia salina* egg extracts against* Pseudomonas aeruginosa*, which is the primary bacterium involved in burn infections. Burn-associated infections caused by* Pseudomonas aeruginosa* are among the most severe infections, causing major delays in burn patient recovery and potential death [48–50]. To analyze the antibacterial activity of various fractions against* Pseudomonas aeruginosa*, we cultured the bacterium on LB agar plates and used the paper disc method [21]. Fifty microliters of 2 *μ*g/*μ*l stock solutions of the extracts was slowly absorbed into the sterilized paper disc and adhered to the surface of the plate. 1% silver sulfadiazine was used as a standard. After culturing for 24 h in an incubator at 37°C, the clear zone around the disk was measured and antibacterial activities were analyzed and compared. We observed that the hexane fraction showed the greatest clear zone (13 mm), which was very close to that of the standard (14.3 mm) (Figure 7). The clear zones of ethyl acetate, chloroform, water, and n-butanol were 10 mm, 9 mm, 8 mm, and 9 mm, respectively (Figure 7). These data suggested that* Artemia salina* egg extracts compounds with antibacterial activity against* Pseudomonas aeruginosa*, which indicates that these extracts may have potential for treatment of burn-associated infections after further purification.

## 4. Conclusion

The current study explored the activity of* Artemia salina* egg extracts against cancer cell lines and* Pseudomonas aeruginosa*.* Artemia salina* egg extracts exhibited potent inhibitory activity against HCT116 and B16-F10 cells. The hexane and chloroform fractions potently decreased the viability of both cell lines. Collectively, 14 compounds were identified from the methanolic crude extract, and the hexane, and chloroform fractions, by GC-MS, many of which were previously associated with anticancer, antioxidant, antimycobacterial, opioid receptor antagonist, antifungal, and anti-inflammatory activities.* Artemia salina* egg extracts induced apoptosis in the HCT116 and B16-F10 cells lines and showed antibacterial activity against* Pseudomonas aeruginosa*. The hexane fraction exerted the strongest antibacterial activity. Therefore, the hexane and chloroform fractions of* Artemia salina* eggs may provide potential therapeutic benefit for the treatment of colorectal cancer, skin cancer, and burn-associated infections.

## Figures and Tables

**Table 1:** Major compounds identified in the methanolic crude extract and the hexane and chloroform fractions of *Artemia salina* eggs. N.D: not determined.

Methanolic crude extract								

S.No	name of compound	nature of compound	molecular formula	molecular weight (g/mol)	RT (min)	area (%)	% of total	established activity

1	Dibutyl phthalate	phthalate	C_16_H_22_O_4_	278.34348	28.932	0.96	0.957	anticancer, antibacterial [25–27, 29]

2	9-octadecenoic acid, methyl ester	unsaturated fatty acid methyl ester	C_19_H_36_O_2_	296.4879	31.592	0.65	0.648	antioxidant, anticancer [28, 30]

3	Oleic acid	fatty acid	C_18_H_34_O_2_	282.4614	32.275	1.97	1.969	antitumor, antibacterial [31–34]

4	di(2-ethylhexyl)phthalate	phthalate	C_24_H_38_O_4_	390.55612	41.927	2.15	2.152	anti-leukemic [37]

Hexane fraction								

S.No	name of compound	nature of compound	molecular formula	molecular weight (g/mol)	RT (min)	area (%)	% of total	established activity

1	Phenol, 2, 4-bis(1, 1-dimethylethyl)	phenol	C14H22O	206.32388	19.791	0.03	0.034	antifungal, antibacterial [42, 43]

2	2-cyclohexen-1-one, 2, 4, 4-trimethyl-3-(3-oxo-1-butenyl)	ketone	C_13_H_18_O_2_	206.2808	23.672	0.15	0.057	anti-inflammatory [44]

3	1-mercapto-2-heptadecanone	thiol	C_17_H_34_OS	286.51626	35.623	0.80	0.800	N.D

4	Methyl 4-(7,7-dimethyl-2,5-dioxo-1,2,3,4,5,6,7,8-octahydroquinolin-4-yl)benzoate	quinoline	C_19_H_21_NO_4_	327.38	38.675	0.53	0.527	N.D

5	Methyl (E)-4′-ethyldiene-3-oxo-2a^1^,4′,5′,8a′-tetrahydro-2′H,7′H-spiro[indoline-2,8′-[3,7]methanofuro[4,3,2-hi]indolizine]-2a′(3′H)-carboxylate	indole	C_21_H_22_N_2_O_4_	366.42	44.520	0.07	0.070	N.D

Chloroform fraction								

S.No	name of compound	nature of compound	molecular formula	molecular weight (g/mol)	RT (min)	area (%)	% of total	established activity

1	Indole-3-carboxaldehyde	indole	C_9_H_7_NO	145.15798	26.698	0.25	0.258	antimycobacterial and anticancer [38]

2	Gancidin W	pyrazine	C_11_H_18_N_2_O_2_	210.27282	28.661	0.46	0.461	antimalarial [45]

3	Naloxone	thebaine derivative	C_19_H_21_NO_4_	327.37434	38.534	1.55	1.555	opioid receptor antagonist, anticancer [39, 40]

4	methyl 1,2,3,4,4a,9,10,10a-octahydro-7-isopropyl-1,4a-dimethylphenanthrene-1-carboxylate	phenanthrene carbaldehyde	C_20_H_28_O_2_	314.22	39.248	1.95	1.947	anticancer [41]

5	Cholest-5-en-3-ol (3.beta.)	cholesterol	C_27_H_46_O	386.65354	47.762	7.20	7.201	N.D

## Data Availability

The data used to support the findings of this study are available from the corresponding author upon request.

## Supplementary Materials

Supplementary Materials Supplementary Table: GC/MS conditions used for analysis and identification of Artemia salina eggs extracts.
