# Supplementary material for: Methanolic Extract of Artemia salina Eggs and Various Fractions in Different Solvents Contain Potent Compounds That Decrease Cell Viability of Colon and Skin Cancer Cell Lines and Show Antibacterial Activity against Pseudomonas aeruginosa
Source: Evid Based Complement Alternat Med. 2019 May 6;2019:9528256. doi: 10.1155/2019/9528256 (PMC6526577; doi:10.1155/2019/9528256)
Supplement: Supplementary Materials — Supplementary Table: GC/MS conditions used for analysis and identification of Artemia salina eggs extracts. [file 9528256.f1.docx]

Supplementary Table. GC/MS conditions used for analysis and identification of Artemia salina eggs extracts.

| Equipment | Agilent (7890B-5977B GC/MSD) |
| --- | --- |
| Column | J & W 122-5532DB-5MS, Capillary GC Column (30m×0.25mm i.d. 0.25 μm film thickness) |
| Oven Equilibration Time | 0.5 min |
| Max Temperature | 350 °C |
| Oven Program | 70 °C for 1 min then 5 °C/min to 300 °C for 20 min |
| Carrier gas | He (99.999%); head pressure of 8.80 psi |
| Run Time | 67 min |
| Total flow | 24 ml/min |
| Injection volume | 1 µl |
| Injector temperature | 200 °C |
| Ionizing voltage | 70 ev |
